# The Impact of the Microbiome on Resistance to Cancer Treatment with Chemotherapeutic Agents and Immunotherapy

**DOI:** 10.3390/ijms23010488

**Published:** 2022-01-01

**Authors:** Aneta Sevcikova, Nikola Izoldova, Viola Stevurkova, Barbora Kasperova, Michal Chovanec, Sona Ciernikova, Michal Mego

**Affiliations:** 1 Department of Genetics, Cancer Research Institute, Biomedical Research Center of the Slovak Academy of Sciences, Dúbravská Cesta 9, 845 05 Bratislava, Slovakia; aneta.sevcikova@savba.sk (A.S.); n.izoldova8@gmail.com (N.I.); viola.stevurkova@savba.sk (V.S.); 2 Department of Genetics, Faculty of Natural Sciences, Comenius University, 842 15 Bratislava, Slovakia; 3 Department of Oncohematology, Faculty of Medicine, Comenius University, Bratislava and National Cancer Institute, 833 10 Bratislava, Slovakia; barbora.kasperova@nou.sk; 4 2nd Department of Oncology, Faculty of Medicine, Comenius University, Bratislava and National Cancer Institute, 833 10 Bratislava, Slovakia; michal.chovanec1@gmail.com (M.C.); misomego@gmail.com (M.M.)

**Keywords:** microbiome, chemotherapy, immunotherapy, treatment resistance, microbiota modulations, probiotics, fecal microbiota transplantation

## Abstract

Understanding the mechanisms of resistance to therapy in human cancer cells has become a multifaceted limiting factor to achieving optimal cures in cancer patients. Besides genetic and epigenetic alterations, enhanced DNA damage repair activity, deregulation of cell death, overexpression of transmembrane transporters, and complex interactions within the tumor microenvironment, other mechanisms of cancer treatment resistance have been recently proposed. In this review, we will summarize the preclinical and clinical studies highlighting the critical role of the microbiome in the efficacy of cancer treatment, concerning mainly chemotherapy and immunotherapy with immune checkpoint inhibitors. In addition to involvement in drug metabolism and immune surveillance, the production of microbiota-derived metabolites might represent the link between gut/intratumoral bacteria and response to anticancer therapies. Importantly, an emerging trend of using microbiota modulation by probiotics and fecal microbiota transplantation (FMT) to overcome cancer treatment resistance will be also discussed.

## 1. Introduction

Recent advances in cancer treatment and clinical implementation of precision medicine have brought about the improvements in both the disease-free survival and quality of life in cancer patients. However, the failure of therapy due to the induced selection of resistant cells within the tumors or unfavorable immune responses are connected with poor patient outcomes and represent a huge challenge. Different mechanisms of drug chemoresistance have been described, related to genetic alterations, DNA damage repair, epigenetic modifications, deregulation of apoptosis, autophagy, and changes in the tumor microenvironment [1,2]. The mechanisms of resistance to immunotherapy are far less defined since complex and patient-dependent interactions in the host immune response are involved. Interestingly, the association between gut and intratumoral microbiota and cancer treatment efficacy represents an emerging trend in microbiome research [3].

The human microbiome influences the hosts’ metabolism via several intrinsic pathways and plays an important role in both shaping and modulating immune system responses. Maintaining healthy gut homeostasis is critical for the host [4], since disturbing the homeostatic crosstalk between the microbiota and the host immune system leads to severe pathological conditions. Animal models, as well as clinical studies, suggest the involvement of gut microbiota in cancer initiation and progression through immune system modulation [5]. At the same time, the ability of the microbiome to potentiate the host immune response against tumors has been reported [6]. Growing evidence from preclinical and clinical findings highlights the fact that the host’s microbiome can affect the potential response to different anticancer modalities, mainly chemotherapy and immunotherapy. This leads to the possibility of modulating the gut microbiota to overcome drug resistance, increase the efficacy of cancer treatment, and restore original healthy microbiota [7]. Still, limited data are available and the microbiome is very likely to have a more significant impact on treatment than expected. In this context, further studies and evaluations may shed more light on microbiome treatment associations.

Herein, we provide a review of the most recent data related to the emerging role of the microbiome in resistance to anticancer therapies, focusing mainly on chemotherapy and immunotherapy with immune checkpoint inhibitors. Importantly, critical findings from animal models, as well as the results from clinical studies supporting the relationship between changes in microbiota composition, the production of microbiota-derived metabolites including short-chain fatty acids (SCFA) and efficacy of cancer treatment will be discussed. Finally, we will outline the potential trend for microbiota modulation by probiotics and fecal microbiota transplantation (FMT) to enhance the response to cancer treatment modalities.

## 2. The Mechanisms of Resistance to Anticancer Therapies

There are several mechanisms, including suppression of programmed cell death, epigenetic changes, altered gene amplification, and DNA breaks repair, which can lead to anticancer drug inactivation [8].

ATP-binding cassette (ABC) transporter proteins, also known as energy-dependent efflux pumps, represent the mechanism related to the drug efflux. These specific transporters consist of two cytoplasmatic and two transmembrane regions [9]. According to the findings, the human body contains a set of 48 ABC transporters divided into seven families (ABCA-ABCG) [10,11]. Three members including ABCB1, ABCC1, and ABCG are implicated in drug transport mechanisms and reduce the accumulation of anticancer drugs [12]. ABCB1, also known as P-gp, is a protein responsible for pumping out anticancer agents such as daunorubicin, doxorubicin, taxol, vinblastine, and vincristine from the plasma membrane to the extracellular space [13]. P-gp is widely expressed in different types of cancer including colon, liver, lung, and rectum, which leads to the reduction of treatment efficacy [14,15]. Anticancer drugs such as anthracyclines, camptothecins epipodophyllotoxins, methotrexate, mitoxantrone, and vinca alkaloids are considered to be substrates for transport by multidrug resistance-associated protein 1 (MRP1/ABCC1) transporter. Yin et al. showed that ABCC1 has an impact on cancer treatment and reduces the efficacy of therapy response [16]. Furthermore, ABCG2 transporter plays a role in resistance to anthracyclines and mitoxantrone in breast cancer cells [17].

Chemotherapy and radiotherapy induce cancer cell death mainly through DNA damage and the presence of genotoxic agents in chemotherapeutic drugs block DNA synthesis during proliferation [18]. However, cancer cells can trigger the repair of damaged DNA by removal of chemotherapy-induced DNA lesions leading to resistance to anticancer therapy and increased cancer cell survival [19]. Cisplatin-mediated DNA damage is repaired by several DNA repair systems including homologous recombination (HR), mismatch repair (MMR), nonhomologous end joining (NHEJ), and nucleotide excision repair (NER). On the other hand, the mechanism of mutagenic translesion synthesis allowed the tolerance of DNA damage after genotoxic chemotherapy [20,21]. NER represents the main pathway for the removal of DNA damage induced by platinum-based derivates (oxaliplatin and cisplatin) [22]. Rosell et al. documented a significant correlation between overexpression of DNA excision repair protein (ERCC-1), DNA repair endonuclease (XPF), and a poor response to platinum-based chemotherapy [23]. Recently involved inhibitors of DNA repair proteins might increase sensitivity to platinum drug-mediated cancer cell death.

Gene amplification and the overexpression of oncogenes associated with resistance to anticancer therapy are observed in 10% of all cancer cases [24]. In addition to genetics, epigenetic modifications are related to resistance to antitumor drugs through increased DNA repair, efflux of anticancer drugs, and impaired cell death [25]. Epigenetic changes including DNA methylation and histone modifications (acetylation and methylation) result in gene expression alterations [26]. During acetylation, chromatin conformation is altered by histone acetyltransferases (HATs) and histone deacetylases (HDACs). DNA methylation status allows transferring a methyl group to CG dinucleotides localized in CpG islands within the promoter gene region. Hypermethylation of tumor suppressor genes leads to gene silencing while hypomethylation of oncogenes is associated with gene overexpression [27,28]. Demethylated promoter of multi-drug resistance 1 (*MDR1*) gene is related to decreased accumulation of therapeutic agent in tumor cells [29]. Currently, several approved epi-drugs are available including histone deacetylase inhibitors (HDACi)–belinostat, panobinostat, romidepsin, and vorinostat [30] as well as DNA methylation inhibitors (DNMTi)—azacitidine and decitabine [31].

The intratumoral microenvironment represents a critical factor inducing resistance to anticancer therapy, so a combination of drug therapies focusing on different subpopulations of cancer cells within the tumor is required for their successful eradication [32]. Besides the cancer cells, immune cells, fibroblasts, and stromal cells can also be found within the tumor microenvironment, contributing to drug resistance and cancer progression [33]. Cancer-associated fibroblasts (CAFs) induce treatment resistance via the secretion of proteins, exosomes, and extracellular matrix (ECM) remodeling factors [34]. In hematological malignancies, mesenchymal stem cells (MSCs)-related activation of the CXCL12/CXCR4 pathway followed by reduced activity of caspase 3 contribute to therapy resistance [35]. In addition, MSCs can transform into cancer stem cells (CSCs) leading to increased chemoresistance [36].

**Figure 1:**
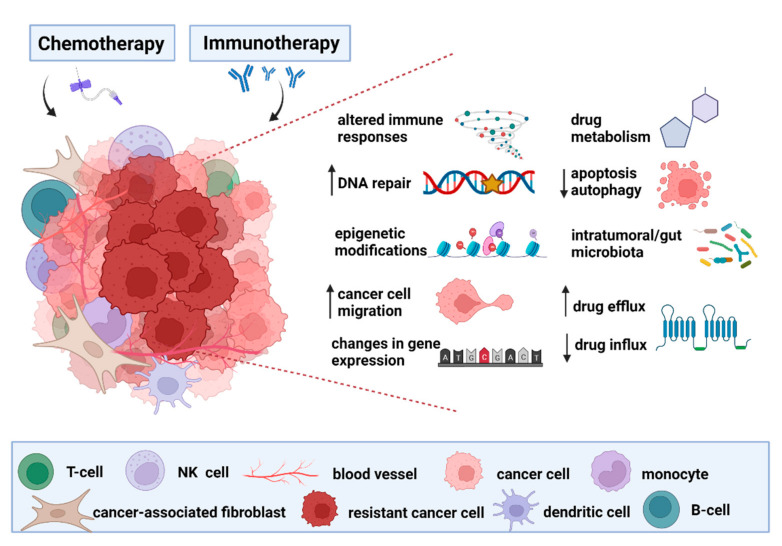
The proposed mechanisms of cancer treatment resistance. The altered expression of well-studied transmembrane proteins known as transporters contributes to a low influx or high efflux of chemotherapeutics, leading to a decreased level of intracellular drug delivery. Moreover, increased metabolism of chemotherapeutic agents can result in the breakdown of the molecules followed by reduced efficacy of anticancer therapy. Epigenetic modifications can have a role in the development of therapy resistance via two main mechanisms, including histone modification (methylation and acetylation) and DNA methylation, which correlate with tumorigenesis and subsequent therapy resistance. Reduced apoptosis and autophagy, as possible mechanisms of cancer defense against therapy, are markers of therapy resistance because of reduced cancer cell death. Epithelial–mesenchymal transition-related pathways contribute to treatment resistance and formation of metastatic cancer cells through decreased expression of cell adhesion molecules and improved cell motility. Chemotherapy aims to induce DNA damage, but there is a potential option to reverse the mechanism of DNA damage through increased expression of repair proteins that may confer drug resistance. Observed changes in gene expression of tumor suppressor genes and oncogenes dramatically influence the activity of target genes, so there is a correlation between genomic alterations and resistance to cancer treatment. If the tumor microenvironment develops some specific mechanisms of resistance, then the adaptive and innate immune response is unable to destroy the tumor cells. Importantly, accumulating evidence reveals an emerging role of altered gut microbiota and its metabolic activity in the resistance to chemotherapeutic agents and immunotherapy.

Increasing evidence support the role of the gut microbiome in modulating the response to anticancer therapies [37,38]. Altered composition of intratumoral/gut microbiota together with other mechanisms can influence the resistance of cancer cells to administered therapy (Figure 1). Importantly, a deep understanding of the complex relationship between the gut microbiota composition and previously described mechanisms of resistance to anticancer therapy may be the key to designing new strategies for improved treatment efficacy in the future.

## 3. Human Gut Microbiome

Trillions of bacteria inhabit the human gut ecosystem and mounting research has revealed the mechanisms of how the gut microbiota influences the host in health and disease. The gut microbiome represents the collection of intestinal microorganisms including bacteria, archaea, viruses, and fungi, together with their overall genetic material. During the last 30 years, progress in sequencing methods has generated data regarding the composition of the healthy gut microbiome, showing that *Bacteroidetes*, *Firmicutes*, and *Proteobacteria* are dominant bacterial phyla. Moreover, other microorganisms including archea, eukaryotic organisms, viruses, and fungi significantly contribute to the stability and diversity of the human gut [39,40]. The comprehensive metagenomics approach provides information on how individual bacterial species can affect the host’s health [41,42,43,44].

The gut is a producer of intestinal mediators which can enter the blood circulation, and affect the vital internal organs such as the brain and liver [45]. Due to its interactions with microbiota, the intestinal epithelium plays a role in recognition of specific bacterial ligands (lipopolysaccharide, lipoproteins, flagellin) allowing the tolerance to commensal bacteria which form the intestinal symbiotic ecosystem [46,47]. Disruption of gut microbiota leads to an abnormal immune response against invasive and inflammation-inducing bacteria. The recognition of microbial pathogen-associated molecular patterns (PAMPs) from translocated bacteria allows activation of Toll-like receptor (TLR) signaling pathway, and triggers oxidative stress and inflammation [48,49,50]. In addition, gut dysbiosis is associated with the development of many intestinal disorders including Crohn’s disease, antibiotic-associated diarrhea, inflammatory bowel disease, and increased risk of gastrointestinal malignancies [51,52,53,54]. Recently, the link between gut microbiome and late effects of anticancer therapies has been proposed [55].

## 4. The Relationship between Microbiome and Resistance to Chemotherapy

The successful use of systemic chemotherapy dates back to the 1940s, when nitrogen mustard proved to be an effective alkylating agent in the treatment of malignant lymphoma [56]. Several cytotoxic drugs that significantly improve cancer treatment and patient survival have been introduced in the last decades. However, the occurrence of adverse effects and acquired drug resistance represent the main challenges in recently administered chemotherapeutical regimens [37]. The microbiota-derived metabolic activation of some azo prodrugs was initially described almost 60 years ago [57]. The gut microbiota co-develops with the host, playing a role in the interface of antitumor and carcinogenic metabolic, inflammatory, and immune pathways [58]. Alexander et al. proposed the TIMER mechanism (Translocation, Immunomodulation, Metabolism, Enzymatic degradation, Reduced diversity and ecological variation), explaining the key processes by which the intestinal microbiota affects the efficacy of the chemotherapeutic agents [59]. 

A pilot study concerning the association between the gut microbiome and therapeutic responses to neoadjuvant chemoradiotherapy (nCRT) revealed different relative abundances of several bacteria taxa before and after nCRT in rectal cancer patients. Similarly, differences in microbiota composition between responders and non-responders have been identified, showing *Shuttleworthia* enrichment in responders while microbiota of non-responders was characterized by a higher abundance of *Clostridiales* [60]. Recently, metagenomic analysis of samples from eight different cancer types described the baseline gut microbiome signatures predicting treatment outcome of cytotoxic or targeted chemotherapy, immunotherapy, or a combination of anti-cancer treatments. Based on microbial differences between responders and non-responders, a positive correlation between *Bacteroides ovatus*/*xylanisolvens* and treatment efficacy was identified by machine learning and proved by oral gavage in mice bearing lung cancer [61].

### 4.1. Platinum-Based Derivates

The antineoplastic mechanism of platinum-based chemotherapeutics (oxaliplatin and cisplatin) involves the formation of intra-stranded DNA adducts, inhibiting DNA replication and activating mitochondrial signaling pathways that cause cell death [62]. Iida et al. reported that a group of antibiotic-treated and germ-free (GF) mice did not respond correctly to platinum derivatives, showing insufficient production of reactive oxygen species related to anti-cancer effects of selected drugs [63]. Moreover, the genes responsible for monocyte activation and differentiation were inhibited after antibiotic administration. After oxaliplatin treatment, the proinflammatory genes were reduced in GF animals, suggesting the importance of inflammation for anticancer treatment [49]. In colorectal cancer (CRC) patients, *Fusobacterium nucleatum* was shown to play the role in oxaliplatin chemoresistance through the activation of the innate immune system. According to the results, induced autophagy, mediated via microRNA (miR-4802 and miR-18a*) downregulation led to oxaliplatin resistance in vitro [64].

### 4.2. Cyclophosphamide

The relationship between microbiota composition and therapeutic efficacy in cyclophosphamide (CTX)-treated murine model has been monitored [65]. Stimulation of anti-tumor immune responses through a variety of immunological pathways, supporting Th1 and Th17 cells to control cancer growth, represents the main mechanism of CTX antineoplastic effects [59,66]. As shown by Viaud et al., the alkylating agent CTX significantly altered the microbiota composition of the small intestine leading to the reduction in the abundance of bacterial species from *Firmicutes* phylum (*Roseburia*, *Coprococcus*, *Clostridium* cluster *XIVa*, *unclassified Lachnospiraaceae*) as well as lactobacilli and enterococci in mice bearing subcutaneous melanomas and sarcomas [65]. Additionally, the microbial barrier of the small intestine was more permeable to Gram-positive bacteria *(Lactobacillus johnsonii*, *Lactobacillus murinus*, *Enterococcus hirae)* leading to their translocation from the gut into the lymphoid organs. Translocated bacteria induced the generation of pathogenic T helper 17 cells and immune response against the tumor. Importantly, antibiotic-treated mice bearing tumors were resistant to CTX action [65]. Daillere et al. confirmed the key bacterial species involved in the immunomodulatory effects of CTX, showing the Gram-negative microorganism *Barnesiella intestinihominis* plays an anticancer immunomodulatory role in the colon. Interestingly, CTX-mediated antitumor effects were restored by oral administration of *Enterococcus hirae*. This finding highlights the importance of reconstituting the optimal microbiota diversity by genera *Enterococcus* and *Barnesiella*, to optimize responses to alkylating agents [67].

### 4.3. Gemcitabine

Gemcitabine is a nucleoside analog used to treat metastatic pancreatic, breast, ovarian, or lung cancer [68]. A modification in the structure of chemotherapeutical drugs including gemcitabine, fludarabine, cladribine, and CB1954 by bacteria was confirmed using high-performance liquid chromatography and mass spectrometry [69]. Moreover, murine colon cancer model CT26 revealed the chemoresistance to gemcitabine and increased cytotoxicity of CB1954 after intratumoral administration of *E. coli*, documenting the ability of bacteria to metabolize chemotherapeutics while affecting their activity and local concentration [69,70].

Geller et al. found that *Gamaproteobacteria* expressing a long form of cytidine deaminase (CDD) can convert the active form of gemcitabine (2′2′-difluorodeoxycytidine) into its inactive form (2′2′-difluorodeoxyuridine) in colon cancer models [71]. Since pancreatic ductal adenocarcinoma (PDAC) responds poorly to treatment with traditional chemotherapeutic agents due to the phenomenon of intrinsic or acquired drug resistance [72], a better understanding of drug resistance mechanisms is needed. The presence of pancreatic intratumoral *Gammaproteobacteria* (*Enterobacteriaceae* and *Pseudomonadaceae* families) has been detected in human PDAC samples, pointing at their potential role in treatment efficacy [71]. Moreover, antibiotic treatment with ciprofloxacin has been shown to overcome gemcitabine resistance [71,73]. The resistance to gemcitabine may also be associated with the presence of *Mycoplasma hyorhinis* and its ability to encode CDD and disrupt the cytostatic activity of chemotherapeutic agent [74]. Elevated levels of oral pathogens *Agregatibacter actinomycetemcomitans* and *Porphyromonas gingivalis*, which may affect resistance to chemotherapy by expressing CDD, have also been observed in patients with pancreatic cancer [75]. This observation suggests the ability of bacteria from other tissues to affect the resistance to and efficacy of chemotherapy [76].

In 2018, Panebianco et al. noted a reduction in tumor volume (approximately 35%) at the end of gemcitabine therapy along with the changes in bacterial composition in applied mouse models. Gemcitabine treatment significantly reduced the proportion of the two dominant phyla—*Firmicutes* (*Lachnospiraceae*, *Ruminococcaceae*, *Erysipelatoclostridium*) and *Bacteroidetes* (*Bacteroidales*, *Alistipes*) from 39 to 17% and from 38 to 17%, respectively. In contrast, the bacterial composition shifted in favor of two phyla which are generally minor constituents of the intestinal microbiota—*Proteobacteria* (*Escherichia coli*, *Aeromonas hydrophila*) and *Verrucomicrobia* (*Akkermansia muciniphila*) from 15 to 32% and from 5 to 33%, respectively [77]. Ganesh et al. reported that *Akkermansia muciniphila* exacerbated intestinal inflammation due to its mucolytic activity [78], which could have a negative effect on gemcitabine-treated mice. According to the previous findings, the overgrowth of proteobacteria was associated with intestinal inflammation and the decrease of bacteria from the phyla *Firmicutes* and *Bacteroidetes* was associated with intestinal pathology [79,80,81]. Moreover, gemcitabine-treated mice reported an increased incidence of the infectious organism *Peptoclostridium difficile* compared to untreated animals [77]. As reported in previous studies, overgrowth of *Peptoclostridium difficile* with *Enterobacteriaceae* is a common consequence of chemotherapy [82,83]. Interestingly, a mouse model of pancreatic cancer treated with gemcitabine and bevacizumab suggested a clinical potential for *Salmonella typhimurium* since its positive effect on changes in tumor size leading to tumor shrinkage [84].

### 4.4. Fluoropyrimidine Analogs and Anthracyclines

The enrichment of *Fusobacterium nucleatum*, a well-known pathogenic bacterium [85,86], was observed in stool samples from colorectal adenoma and carcinoma patients compared to healthy controls [87]. Mima et al. showed that relative abundance of *Fusobacterium nucleatum* was associated with worse clinical outcomes in CRC patients [88]. In addition, the relationship between *Fusobacterium nucleatum* together with certain bacterial taxa including the genus *Sutterella* and species *Veillonella dispar*, and the resistance to a chemotherapeutic cocktail containing tegafur (a prodrug of 5-fluorouracil, 5-FU) and oxaliplatin was detected in CRC patients [89]. More recently, *Fusobacterium* was reported to be responsible for chemoresistance to 5-FU and oxaliplatin in patients with CRC via activation of the innate immune system [64]. *F. nucleatum* plays an important role in the colon cancer microenvironment since interaction with the immune cells leads to an increase in tumor-associated neutrophils, dendritic cells, and pro-cancer M2 macrophages, and inhibition of the cytotoxicity of T and NK cells represses the host immune responses [90].

According to the findings, a few bacterial species play a role in the metabolism of anthracyclines, and the ability of *Streptomyces* WAC04685 and *Raoultella planticola* to inactivate doxorubicin by deglycosylation mechanism has been described [91,92].

A chronological summarization of studies dealing with the impact of the microbiome on various chemotherapeutic agents is provided (Table 1).

## 5. Gut Microbiome Shapes the Efficacy of Immunotherapy

**Figure 2:**
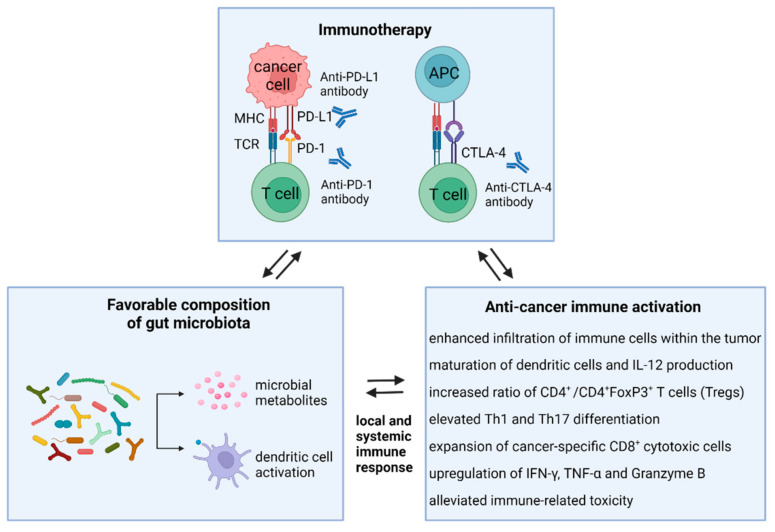
Modulatory effects of favorable gut microbiota on the immune system and immunotherapy efficacy. Microbiota-directed activation of anti-cancer immunity significantly affects the response of cancer patients to immunotherapy with immune checkpoint inhibitors (anti-PD-1, anti-PD-L1, or anti-CTLA4). The host immune response is triggered by microbiota-derived metabolites, like SCFA, and by recognition of bacterial signals with dendritic cells. Subsequently, T cell priming, depending mainly on the cytokine milieu, leads to T cell differentiation into immunosuppressive Treg cells, Th1/Th17 cells, and effector T cells. Th1 CD4^+^ T cell differentiation and activation of CD8^+^ cytotoxic cells result in the production of specific cytokines and tumor killing. Abbreviations: IFN-γ, interferon gamma; TNF-α, tumor necrosis factor α.

Antitumor immunotherapies enhance the host’s immune system to recognize and target cancer cells, as opposed to using cytotoxic treatment with chemotherapeutic agents to directly kill the tumor cells [94]. Since numerous chemotherapy-induced side effects significantly impact patient outcomes, the introduction of immunotherapy represents a critical step in cancer treatment, showing a positive effect on the treatment of melanoma, non-small cell lung cancer (NSCLC), renal cell cancer (RCC), and also hematological malignancies [95]. Importantly, the therapeutic effect of immunotherapy depends on tumor heterogeneity, environmental factors, and the host immune system [96,97,98,99], which is in turn connected with genetic background and also with the gut microbial composition (Figure 2).

### 5.1. Immune Checkpoint Inhibitors

The expression of immune checkpoint proteins CTLA-4 and PD-1/PD-L1 contributes to the protection of healthy body tissues and helps to maintain immune homeostasis [95,100]. However, cancer cells have been shown to exploit these checkpoints to evade the immune system via the activation of a specific PD-1/PD-L1 pathway which induces immune tolerance within the tumor microenvironment [101]. PD-1, expressed on activated immune cells such as B cells, natural killer cells, macrophages, monocytes, and T cells, is considered to have an inhibiting effect on adaptive and innate responses of the immune system [102]. To avoid the elimination of tumor cells by T cells, PD-L1 plays the role of a pro-tumorigenic factor and this ligand is expressed on the surface of cancer cells. The interaction between PD-1 and its ligand triggers the process of immune T-cell inactivation [103,104,105]. CTLA-4 protein is expressed by both CD4^+^ and CD8^+^ T cells. CTLA-4 can bind to CD80 and CD86 ligands on antigen-presenting cells with higher affinity and avidity than homologous CD28 [106,107]. Through these interactions, CTLA-4 further inhibits T cell responses [108]. Targeting the immune checkpoints CTLA-4, PD-1, and PD-L1 help to restore the anti-cancer activity and represents the emerging trend in immunotherapeutic approaches [101,104]. In particular, monoclonal antibody ipilimumab participates in CTLA-4 blockade, allowing it to reactivate T cells and eliminate tumor cells with its ligands [95,109]. Furthermore, durvalumab and atezolizumab act as monoclonal antibodies blocking PD-L1 in cancer patients [110].

Monotherapy with pembrolizumab, designed to block PD-1, has brought higher overall survival in patients with NSCLC but increased heterogeneity in treatment response. Thus, a combination of immunotherapies is suggested, rather than monotherapy, as a novel strategy for enhancing the efficacy of treatment [111,112,113]. Hodi et al. documented a higher clinical benefit in the group of advanced melanoma patients treated with nivolumab together with ipilimumab compared to the patients on nivolumab therapy alone [114]. More recently, the approved cemiplimab and dostarlimab are also effective antibodies targeting the PD-1 signaling pathway [115] and several other PD-1 inhibitors are currently under development [116].

### 5.2. Animal Models Concerning the Role of the Gut Microbiome in Immunotherapy

Mounting evidence from preclinical models and clinical studies emphasize the essential role of host microbiota composition in immunosurveillance and the response to immunotherapy, suggesting a potential for the use of microbiota modulation in overcoming treatment resistance.

Ida et al. showed reduced therapeutic effects of immunotherapy via a combination of TLR9 antagonist and antibody to interleukin-10R (IL-10R) in mice treated with broad-spectrum antibiotics or GF animals [63]. Optimal responses to cancer treatment required an intact commensal microbiota that mediated therapeutic effects by modulating myeloid-derived cell functions in the tumor microenvironment. Microbiota disruption decreased the response of subcutaneous tumors to CpG-oligonucleotide immunotherapy via low levels of tumor necrosis factor (TNF). Tumor-infiltrating myeloid-derived cells responded poorly to therapy, resulting in lower cytokine production and tumor necrosis after CpG-oligonucleotide treatment [63]. Accordingly, an existing link between gut microbiota and the increased efficacy of immunotherapy with immune checkpoint inhibitors was documented [96,117]. Vetizou et al. demonstrated the relationship between T cell responses specific for *Bacteroides thetaiotaomicron* and *Bacteroides fragilis* and the efficacy of CTLA-4 blockade in animal models as well as in cancer patients. Oral gavage with *B. fragilis*, immunization with *B. fragilis* polysaccharides, or adoptive transfer of *B. fragilis* restored the response to immunotherapy in antibiotic-treated or GF non-responding animals. Furthermore, immunotherapy by ipilimumab has modified the gut microbiome in metastatic melanoma patients at the genus level, leading to a rapid decrease of bacterial species from *Bacteroidales* and *Burkholderiales* with a relative abundance of *Clostridiales.* The effect of anticancer therapy was improved by fecal transplantation, documenting the key role of *Bacteroidales* in the immunostimulatory effects of CTLA-4 blockade [117]. On the contrary, Chaput et al. found stable gut bacterial diversity without significant changes in the presence of *Bacteroidetes* and *Firmicutes* in patients with metastatic melanoma receiving ipilimumab treatment [118]. Moreover, the administration of antibiotics prior to immunotherapy did not alter dominant bacterial species within the microbiota. These discrepancies reflect the potential differences in the efficacy of immunotherapy treatment between mice and humans [117,118]. The analysis of tumor size after PD-L1 blockade in genetically similar mice revealed the tumor reduction in non-responder animals after receiving feces from immunotherapy responders. Accordingly, orally administered *Bifidobacterium* had a positive impact via increased response to immunotherapy [96].

To determine the relationship between gut microbiota and efficacy of anticancer therapy, antibiotic (ATB)-treated BALB/c mice were supplemented with FMT from patients with RCC. After inoculation of mice with renal cancer cells, treatment with a combination of monoclonal antibodies against PD-1 and CTLA-4 was administered. In contrast to FMT from non-responding patients, the transfer of stool from responders helped to restore anticancer efficacy of immunotherapeutic PD-1 and CTLA-4 blockade [119]. Xu et al. performed an analysis concerning the correlation between administration of different antibiotics prior to the immunotherapy initiation and PD-1 antibody immunotherapy efficacy in a colorectal carcinoma model of CT26 tumor-bearing mice. Broad-spectrum antibiotics caused changes in taxonomic gut composition and the animals did not respond to PD-1 blockade. The enrichment of *Akkermansia muciniphila* and *Prevotella* spp. increased the benefit of anti-PD-1 therapy by altering glycerolipid metabolism while the prevalence of *Bacteroides* interfered with poor response to immunotherapy [120]. Similarly, reduced efficacy of immunotherapy in specific pathogen-free (SPF) mice bearing colorectal tumors treated with broad-spectrum antibiotics was described in a study by Mager et al. [121]. Monocolonization of GF animals with *Bifidobacterium pseudolongum*, *Lactobacillus johnsonii* or *Olsenella* spp. helped to improve the potency of immunotherapy, in contrast to colonization with *Colidextribacter* or *Prevotella* spp. Importantly, a higher amount of bacterial inosine was detected in the serum of GF mice supplemented with *Bifidobacterium pseudolongum* [121].

### 5.3. Clinical Studies Reveal the Role of Gut Microbiota in Immunotherapy Response

Shotgun sequencing of fecal samples from NSCLC and RCC patients has identified the relationship between a relative abundance of *Akkermansia muciniphila* and clinical response to immunotherapy. The results showed that *Akkermansia* was over-represented only in fecal samples from good responders. Oral supplementation with *Akkermansia muciniphila*, either alone or in a combination with *Enterococcus hirae*, after recolonization of GF or ATB-treated SPF mice with feces from non-responders led to the restoration of the efficacy of PD-1 blockade in an interleukin-12-dependent manner [119]. Gopalakrishnan et al. performed a metagenomic analysis of 112 melanoma patients detecting a higher bacterial diversity and enrichment of *Faecalibacterium* species belonging to *Clostridiales* in the gut microbiome of patients responding to PD-1 blockade. On the contrary, fecal samples from poor responders were enriched by *Anaerotruncus colihominis*, *Bacteroides thetaiotaomicron*, and *Escherichia coli.* The presence of *Faecalibacterium* genus correlated with a longer time of survival without progression after anti-PD-1 treatment, while patients with higher levels of *Bacteroidales* showed a reduced survival rate [122]. Similarly, a study of 42 metastatic melanoma patients by Matson et al. found differences in microbiota composition between patients responding to PD-1 blockade and non-responders. Fecal samples from responders were abundant in species *Bifidobacterium adolescentis*, *Bifidobacterium longum*, *Collinsella aerofaciens*, *Enterococcus faecium*, *Klebsiella pneumoniae*, *Parabacteroides merdae*, *Veillonella parvula.* On the other hand, two bacterial species *Roseburia intestinalis* and *Ruminococcus obeum* were highly represented within the microbiota of poor responders [123]. Metastatic melanoma patients treated with ipilimumab enriched in *Bacteroidaceae*, *Rikenellaceae*, and *Barnesiellaceae* have been shown to be resistant to ipilimumab-caused colitis, and the presence of *Bacteroidetes* can increase differentiation of T regulatory cells [124]. The analysis of stool samples from 38 patients with solid tumors receiving anti-PD-1 treatment found the differences in gut microbiota diversity between responders and non-responders supporting the fact that higher diversity can enhance immunotherapy response. A significant abundance of bacterial family *Ruminococcaceae* belonging to the *Clostridiales* was identified in the stool samples from patients who have responded to treatment [125]. In a study by Botticelli et al., differences in gut microbiota composition between nivolumab treated NSCLC patients and healthy controls were reported. In fecal samples of responders, *Akkermansia muciniphila*, *Bifidobacterium longum*, *Faecalibacterium prausnitzii*, *Peptostreptococcus*, *Propionibacterium acnes*, *Staphylococcus aureus*, *Veillonella parvula* were more abundant while *Dialister*, *Ruminococcus bromii*, and *Sutterella* were less presented [126]. *Faecalibacterium* and *Roseburia* were increased in patients with metastatic RCC responded to nivolumab compare to the patients with disease progression [127].

The association between anti-PD-1 immunotherapy and gut composition was also observed in a small group of patients with hepatocellular carcinoma. The authors described no dysbiosis at the baseline (prior to therapy) and the presence of three dominant phyla—*Bacteroidetes*, *Firmicutes*, and *Proteobacteria*—in feces from both responders and non-responders. The composition of dominant phyla was unchanged in responder samples during the treatment. However, elevated levels of *Escherichia coli* were detected in non-responder samples along with continuing treatment. Similar to previous studies, commensals *Akkermansia muciniphila* and *Ruminococcaceae* spp., inhibiting the increased permeability through the intestinal barrier, were identified in responder samples [128]. Recently, Salgia et al. performed a prospective study of fecal samples from 31 patients with metastatic RCC prior to immunotherapy initiation (either with nivolumab or ipilimumab plus nivolumab). The gut microbiome profiling revealed that the increased presence of *Akkermansia muciniphila* was connected with patients´ clinical benefit from immune checkpoint blockade [129].

Importantly, patients with dominant members from *Firmicutes* phylum reported longer overall survival but an increased risk for ipilimumab-induced colitis. On the other hand, *Bacteroidetes*-enriched patients showed the absence of immunotherapy-induced colitis even these patients were characterized as poor antitumor responders [118]. A recent study involving 27 metastatic melanoma patients undergoing immunotherapy found that a higher diversity of the gut microbiome and enrichment of *Coprococcus eutactus*, *Faecalibacterium prausnitzii*, *Lachnospiraceae bacterium 3 1 46FAA*, *Prevotella stercorea*, *Streptococcus anginosus*, and *Streptococcus sanguinis* in pre-treatment stool samples was associated with longer progression-free survival. In contrast, reduced survival without progression was related to *Bacteroides dorei*, *Bacteroides massiliensis*, *Bacteroides ovatus*, *Blautia producta*, *Lachnospiraceae bacterium 5 1 57FAA*, and *Ruminococcus gnavus*. For patients with longer survival, the pathway for biosynthesis of L-isoleucine by *Coprococcus eutactus* was characteristic, while pathways related to shorter survival were specific for synthesis of 6-hydroxymethyldihydropterin diphosphate, coenzyme A, flavin, guanosine nucleotides, pantothenate, pyridoxal 5-phosphate, and for degradation of L-rhamnose [130].

A study of patients with NSCLC, urothelial carcinoma, and RCC on PD-1/PD-L1 immunotherapy revealed a reduced survival without progression, as well as overall survival in patient groups receiving oral antibiotic therapy 8 weeks prior or 4 weeks after the immunotherapy. These results suggested that efficacy of cancer therapy might be affected by antibiotic-mediated changes in gut microbiota composition followed by dysbiosis [119]. Pinato et al. confirmed that patients on antibiotics prior to immunotherapy had poor treatment response and consequently decreased overall survival. In many cases, these patients had to interrupt therapy, and died due to disease progression [131]. Antibiotic usage in 239 NSCLC and 121 RCC patients treated with anti-PD-1 monotherapy alone or in combination confirmed that the use of antibiotics before initiating immunotherapy was associated with reduced benefit from anti-PD-1 blockade in both groups of treated patients. Overall survival and progression-free survival were significantly shortened, suggesting that modulation of gut microbiota by favorable species can aid recovery of antibiotic-caused dysbiosis [132]. In addition, the efficacy of nivolumab was successfully restored in non-responding mice after FMT from responding RCC patients or by *Akkermansia muciniphila* and *Bacteroides salyersiae* [133].

The critical findings from preclinical and clinical studies concerning the relationship between gut microbiome and efficacy of immunotherapy are summarized chronologically in Table 2.

## 6. Microbiota-Derived Short-Chain Fatty Acids and Cancer Therapy

The gut microbiome influences immune response both directly and through microbiota-derived metabolites [137]. As recently reviewed, gut microbial metabolites can be divided into three groups according to whether they are (i) produced by gut bacteria from dietary components, (ii) de novo synthesized by gut bacteria, or (iii) produced by the host and modified by gut bacteria [138]. Propionate, acetate, and butyrate are among the main SCFA generated by gut microbiota from non-digestible and fermentable carbohydrates [139], playing a role in gut barrier integrity, anti-inflammatory, and immune response and metabolism of lipids, cholesterol, and glucose [140]. While representatives from *Bacteroidetes* phylum are the main producers of acetate and propionate, *Firmicutes* phylum is typical for butyrate production [141]. SCFA formation represents the key mechanism by which a high fiber diet exerts anti-cancer influences [142]. The intestinal microbiome affects the level of expression of genes encoding enzymes involved in SCFA metabolism. Cherbuy et al. documented that butyrate-producing microorganisms increased the expression of mHMGCoA synthase (mitochondrial 3-hydroxy 3-methyl glutaryl CoA) responsible for the biosynthesis of ketone bodies from butyrate. The absence of butyrate-producing microbiota resulted in low butyrate metabolism due to the low expression of the enzymes involved in this process [143].

### The Role of Butyrate in Cancer Prevention and Treatment Efficacy

Among all SCFA, special attention is paid to butyrate due to its multiple beneficial effects at both intestinal and extraintestinal levels [144]. Key butyrate producers include *Faecalibacterium prausnitzii* (*Clostridium leptum* cluster) and *Eubacterium rectale*/*Roseburia* spp. (*Clostridium coccoides* cluster) [145]. According to several human studies, lower levels of butyrate-producing bacteria were detected in the gut microbiota of CRC patients compared to healthy participants [146,147], suggesting the role of butyrate in cancer prevention. Additionally, a negative correlation between fecal butyrate levels and colorectal tumor size has also been found [148,149]. On the other hand, some animal models and human studies have demonstrated the promoting effect of butyrate on colorectal carcinogenesis [150,151]. This butyrate paradox was previously explained by differences in butyrate concentrations [152]. Recent findings by Okumura et al. suggest that a subset of butyrate-producing bacteria may contribute to cellular senescence and colorectal tumorigenesis [153].

A large number of studies have been performed to elucidate the molecular mechanism of the anti-cancer effects of butyrate [154]. According to the findings, butyrate belongs to the group of HDACi [155]. The mechanisms of HDACi action involve changes in the acetylated state of chromatin and non-histone proteins, which are manifested by altered gene expression, cell cycle arrest, induction of apoptosis, inhibition of angiogenesis, and metastasis [156]. Thus, oncogenic signaling pathways in carcinogenic cells could be inactivated by butyrate and other HDACi [157]. These pathways include mitogen-activated protein kinase 1 (MAPK1), which inhibits apoptosis and promotes cancer cell proliferation [158], or small mothers against decapentaplegic homolog 3 (SMAD3) required in the epithelial-mesenchymal transition (EMT) process [159]. For cancer treatment and prevention, the ability of butyrate to de-repress epigenetically silenced genes in cancer cells, including p21, Bcl-2 homologous antagonist/killer (BAK), and to activate them in normal cells is considered to be important [160]. Butyrate can act on primary chemoprevention by transcriptional up-regulation of glutathione S-transferase (GST), protecting cells from genotoxic carcinogens [161,162]. Moreover, the relationship between butyrate and decreased expression of vascular endothelial growth factor (VEGF) and hypoxia-inducible factor α (HIF-1α) has been observed, revealing its antiangiogenic and anti-metastatic effects [163,164]. Kuefer et al. described the effect of butyrate on human prostate cancer cell lines, showing the ability of this SCFA to inhibit growth and induce apoptosis also in non-colonic cell lines [165]. Due to this fact, butyrate is also suggested to be effective in secondary chemoprevention [144].

Since clinical studies have revealed a beneficial clinical response to immunotherapy in melanoma patients with an intestinal microbiota enriched in *Faecalibacterium* and other *Firmicutes* [118,122], the association between gut microbiota composition, the effect of SCFA on the immune system, and the clinical response to ipilimumab have been explored. Interestingly, the results showed that systemic butyrate and propionate limit the antitumor effect of anti-CTLA-4 in mice and patients with metastatic melanoma [166]. Inhibition of glycolysis represents an emerging trend in cancer research [167]. Butyrate serves as a ligand for the GPR109a receptor in cancer cells, regulating tumor growth through the GPR109a signaling pathway [168]. Geng et al. discovered that butyrate significantly suppressed glucose metabolism in colorectal cell lines by reducing the abundance of membrane GLUT1 and cytoplasmic G6PD via the GPR109a-AKT signaling pathway. In addition, combined 5-FU/butyrate treatment increased chemotherapeutic efficacy in CRC cells [154]. Recently, a study on 3D patient-derived CRC organoid culture systems (CRC-PDO) has focused on the potential use of butyrate, propionate, and acetate as effective radiosensitizers, increasing the sensitivity of cancer cells to radiation. Of the three SCFA tested, only butyrate showed suppression of organoid proliferation. An increase in radiosensitivity by increasing FOXO3A transcriptional activity and inhibiting the cell cycle regulation by p21, p57, and GADD45 was documented. Since no adverse effects on normal PDO have been reported, butyrate is thought to exhibit selective antitumor activity on CRC-PDO. This observation suggests that butyrate may protect normal mucosal cells while increasing the effectiveness of radiotherapy [169]. However, before the introduction of butyrate as a radiosensitizer into clinical practice, it is necessary to optimize the dose because its higher concentrations may be toxic to healthy intestinal cells.

## 7. Microbiota Modulation as an Emerging Tool towards Improved Response to Anti-Cancer Therapies

Treatment-induced changes in bacterial composition highlight the possibilities of microbiota modulation to improve the clinical benefit of anticancer therapies [129]. Available data suggest that manipulating the microbiota by probiotics and FMT may enhance the treatment efficacy [113] as well as reduce post-treatment immune-related complications [170]. Additionally, several studies described the relationship between specific diets and response to anticancer therapy.

### 7.1. Probiotics

The use of probiotics in oncology is gaining still more attention [171] and the prevalence of cancer patients reporting probiotic administration is relatively high [172]. Prevention and treatment of intestinal toxicity associated with anticancer therapies together with improved immune responses have been described [173,174,175,176,177]. Recent findings also reveal the potential association of probiotic administration with improved efficacy of therapy.

Cisplatin is helpful in treating patients with lung cancer, but a high risk of drug resistance exists. The analysis of a laboratory murine model with lung cancer confirmed that the size of tumors was significantly reduced after cisplatin treatment. Interestingly, a lesser degree of tumor reduction was revealed in the group of mice treated with cisplatin in combination with an antibiotic cocktail. But in the case that cisplatin-treated mice were supplemented with *Lactobacillus acidophilus*, the tumors were reduced more significantly compared to the animals treated with cisplatin alone. The survival rate of mice with lung cancer was higher in the group treated with cisplatin together with *L. acidophilus* supplementation via the orogastric route, suggesting the anti-tumor effect of well-balanced intestinal microbiota [93,178]. Similarly, a study by Maroof et al. documented that oral supplementation with *L. acidophilus* led to the attenuation of tumor growth in mice bearing breast cancer [179].

Since higher microbial diversity has been noted in long-term survivors of PDAC compared to those with shorter survival [180], many studies have focused on the use of probiotics to improve the prognosis of pancreatic cancer. A combination treatment with gemcitabine together with *Lactobacillus paracasei* improved the efficacy of chemotherapy, showing lower levels of aspartate-aminotransferase (AST) and alanine aminotransferase (ALT) [181]. In addition, the antitumor effect of probiotic-derived ferrichrome (*Lactobacillus casei*) has been observed in 5-FU-resistant pancreatic cancer cells, presumably through p53 upregulation and induction of apoptosis [182]. Iwama et al. reported that the effect of ferrichrome on CRC cells was superior to that of 5-FU or cisplatin [183]. These observations suggest that probiotics exhibit antitumor effects mediated by molecules such as ferrichrome and could therefore be used as antitumor drugs in the future [182].

Frankel et al. performed a detailed microbiome analysis of 39 metastatic melanoma patients treated with different types of immunotherapy, including ipilimumab/nivolumab/pembrolizumab or combined nivolumab plus ipilimumab. Microbiomes of all responder samples were enriched in *Bacteroides caccae* and *Streptococcus parasanguinis*. Additionally, an abundance of *Faecalibacterium prausnitzii* and *Holdemania filiformi* belonging to the *Firmicutes* phylum and *Bacteroides thetaiotamicron* from the *Bacteroidetes* phylum was identified in the fecal microbiome of responders treated with a combination of ipilimumab and nivolumab. In the case of pembrolizumab treatment, elevated levels of *Dorea formicigenerans* were detected in patient feces. However, no significant effect of antibiotic nor probiotic treatment was stated [134].

### 7.2. Fecal Microbiota Transplantation

Transfer of fecal microbiome between two melanoma-bearing mouse models JAX and TAC bearing B16.SIY have been shown to elevate specific tumor lymphocytes and suppress tumor growth [96]. In particular, FMT from JAX animals inhibited the tumor growth and brought the synergic effect with PD-1 blockade [96]. Gut microbiome analysis revealed that the abundance of *Bifidobacterium* spp. promoted the antitumor immunity and facilitates anti-PD-L1 efficacy [96]. Importantly, both the administration of *Bifidobacterium* with or without PD-1 blockade showed the significant antitumor effect via increased interferon gamma (IFN-γ) production, maturation activation, and shift in the function of dendritic cells (DCs) [96].

**Figure 3:**
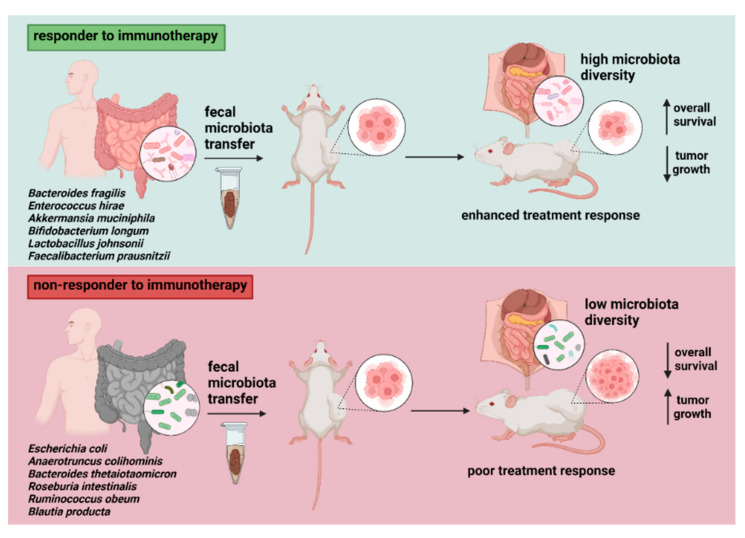
The impact of fecal transfer from responders vs. non-responders on immunotherapy efficacy in tumor-bearing murine models. Cancer patient to mouse models transfer reported improved response to treatment with immune checkpoint inhibitors after FMT from responders, documented by retarded tumor growth and immune activation by elevated levels of CD8^+^ T cells. Donor microbiota enriched by particular bacterial species (most reported are listed) might contribute to microbial alterations in recipient animals, determining the overall treatment effect. Discrepancies related to several bacterial taxa (e.g., *Ruminococcaceae*) showing abundance in both responder and non-responder donor samples suggest the existence of more complex and patient-dependent host–microbiome interactions.

Interestingly, experiments with FMT from cancer patients to mouse models reported enhanced treatment response in animals receiving a fecal transplant from responder donors (Figure 3). In addition, the findings indicated a higher fraction of cells expressing CD45+CD11b+Ly6G+ and a lower level of myeloid cells expressing CD11b+CD11c+ in animals supplemented with FMT from treatment responders. In contrast, recipients of fecal material from poor responders had higher levels of CD4+IL17+ Th17 cells and CD4+FoxP3+Tregs in the spleen, leading to the suppression of anticancer immunity [122]. Riquelme et al. found the ability to differentially modulate pancreatic tumor microbiome and affect tumor growth as well as tumor immune infiltration via human-into-mice FMT from short- and long-term survivals, or control donors. This finding demonstrates the existing cross-talk between gut and tumor microbiome influencing the host immune response [180].

Fecal transplants from three responder and three non-responder patients into recipient GF mice followed by implantation of B16.SIY melanoma cells supported previous findings that the commensal microbiome may have an impact on antitumor immunity. Importantly, improved tumor control, augmented T cell responses, and enhanced efficacy of anti-PD-L1 therapy have been observed in recipients of fecal material from responding patients. Slower tumor growth in two of three mouse cohorts transplanted with fecal material from responders has been detected. On the other hand, two of the three cohorts reconstituted with feces from non-responders reported faster baseline tumor growth. However, the authors concluded that findings from animals usually, but do not always, correlate with clinical response to anti–PD-1 seen in cancer patients [123].

A study of FMT from two donor stool samples enriched with favorable microbiota including *Lachnospiraceae*, *Ruminococcaceae*, and *Veillonellaceae* to a group of 10 nivolumab-refractory metastatic melanoma patients showed partial response in two, while one achieved a complete response [135]. A very recent study on refractory metastatic melanoma patients described the possibility to induce/modulate the immune response to pembrolizumab immunotherapy through FMT from donors via reprogramming the tumor microenvironment in recipients. After FMT, the gut bacterial community altered notably towards donor composition. Responders´ microbiomes were enriched in bacterial phyla *Actinobacteria* and *Firmicutes* while reduced levels of the *Bacteroidetes* phylum were documented. Immunological effect correlated with activated and differentiated CD8+ T cells and lower frequency of myeloid cells expressed interleukin-8 in responders´ samples. According to the study, the use of FMT could improve the response to immunotherapy, although in some patients the absence of immunogenicity in tumor cells, lack of favorable bacterial taxa important for the success of anti-PD-1 therapy, or failure of microbiota transplantation meant that improvements in treatment efficacy were not achieved [136].

### 7.3. Diet and Dietary Components

The impact of diet and dietary components on the composition of the gut microbiome is widely studied [184]. Both ketogenic and high-fiber diets can modulate and reshape gut microbiota, so their use in the cancer treatment approach is gaining still more attention. Preclinical models documented several contradictory findings regarding the anti- and pro-tumorigenic effect of ketogenic diet depending on cancer type, genetic background, tumor model, and specific diet composition [185]. Several studies showed the association of a therapeutic ketogenic diet with hindered tumor growth, longer survival time [186,187], increased sensitivity of tumors to chemotherapy and radiotherapy [188,189,190], reduced metastatic potential [191] and overcoming drug resistance to targeted therapy [192]. On the other hand, mice with BRAF V600E-expressing human melanoma A375 cell xenografts reported increased tumor size after ketogenic diet therapy [193].

According to the finding, high-fiber diets increase the levels of SCFA [194,195]. Low intake of fiber intake resulted not only in reduced microbiota-derived SCFA production but also in the utilization of less-favorable substrates, such as amino acids and host mucins [196,197]. Currently, changes in bacterial diversity after high-fiber diets led to better outcomes from PD-1 blockade therapy and significantly improved progression-free survival in melanoma patients [198]. However, further preclinical and clinical research focusing on safety is highly warranted. In addition, the development of standardized methods, assessments, and outcome measures might be necessary to determine the real impact of specific diets on cancer treatment efficacy.

## 8. Conclusions and Future Directions

Mounting evidence from preclinical and clinical studies highlights the crucial role of microbiota not only in cancer initiation and progression but also in the efficacy of anticancer therapies, mainly chemo- and immunotherapy. The identification of specific bacterial taxa which represent microbial biomarkers linked with enhanced responses to cancer treatment, is the key for the development of microbial-based and microbial-targeted therapies. Importantly, standardization of whole steps in microbiome analyses—including sampling, storage of fecal samples, the choice of experimental design, and bioinformatics tools for determining the microbiota composition—represents the critical issue.

Besides the gut microbiome, bacteria found in tumor samples have also been shown to play an important role in treatment resistance, mainly via modulations of drug metabolism and immune activation. However, detailed molecular mechanisms behind the relationship between tumor and gut microbiome, host immune response, and primary, adaptive, and acquired resistance to cancer treatments are still unclear.

Several issues associated with microbiota-related therapeutic interventions, including safety and feasibility of the approach, the role of prebiotics as well as the impact of diet and food composition, represent important areas of cancer research. Moreover, the selection between administration of well-defined strains in probiotic formula and fecal microbiota transfer remains another unresolved question. Further research should also assess the impact of patients’ genetic background and variable expression of receptor proteins within the gut on microbiota composition. The stability of patients´ gut microbiome and its resistance to perturbation might be implicated in the efficacy and toxicity of anticancer treatment and quality of life.

Comprehensive research aiming at a deep understanding represents a big challenge and can bring benefits for non-responding patients. In the era of precision medicine, evaluation of patients´ gut dysbiosis followed by microbiota modulation-related approaches may provide an emerging trend for optimizing the responses to anticancer therapies and improving outcomes for cancer patients.

## Figures and Tables

**Table 1:** The relationship between gut/intratumoral microbiome and chemotherapy. The table summarizes the major findings from preclinical and clinical studies.

Model	Type of Immunotherapy	Malignancy	Major Findings	Study [Ref.]
mouse feces	cisplatin/oxaliplatin	colon cancer lymphoma melanoma	The effect of antitumor agents was significantly reduced in case of tumor-bearing mice treated with antibiotics. The production of ROS by oxaliplatin was not induced in antibiotic-treated animals, disturbing the efficacy of oxaliplatin-induced DNA damage and apoptosis. The expression of proinflammatory genes was downregulated in the absence of gut microbiota.	Iida et al. 2013 [63]
mouse feces	cyclophosphamide doxorubicin	melanoma sarcoma	The gut barrier of murine models was disrupted after cyclophosphamide treatment, leading to a higher permeability for commensal bacteria such as *Lactobacillus johnsonii*, *Lactobacillus murinus*, *Enterococcus hirae*, and microbiota changes within the small intestine. Antibiotic administration inhibited the effect of cyclophosphamide to cure cancer.	Viaud et al. 2013 [65]
mouse tumor samples	gemcitabine and bevacizumab	pancreatic cancer	Mouse model treated with chemotherapy agents revealed the beneficial effect of *Salmonella typhimurium* A1-R, documented by significantly decreased tumor growth compared to control samples.	Hiroshima et al. 2014 [84]
mouse tumor samples	gemcitabine	breast carcinoma	Antitumor effect of gemcitabine was decreased in mice with *Mycoplasma hyorhinis*-infected murine mammary tumors in comparison with animals bearing unaffected breast tumors.	Vande et al. 2014 [74]
mouse tumor samples	cisplatin	lung cancer	Cisplatin-treated mice receiving antibiotic cocktail reported larger tumors and reduced survival. Both parameters were improved after orogastric administration of *Lactobacillus acidophilus* to lung tumor-bearing mice on cisplatin treatment.	Gui et al. 2015 [93]
mouse tumor samples	gemcitabine CB1954	colorectal carcinoma	According to the results, intratumoral-injected *Escherichia coli* decreased the efficacy of gemcitabine and increased the toxicity of CB1954 in a mouse model with colorectal carcinoma.	Lehouritis et al. 2015 [69]
mouse feces intestinal mucosa	cyclophosphamide	melanoma sarcoma	Both *Enterococcus hirae* and *Barnesiella intestinihominis* have played an important role in antitumor effect of alkylating agents. The reduced effect of chemotherapy with cyclophosphamide in antibiotic-treated mice was compensated by oral gavage of *Enterococcus hirae* which led to a restoration of antitumor activity. On the other hand, *Escherichia coli*, *Lactobacillus johnsonii*, or *Lactobacilli* isolates failed to restore the efficacy of therapy.	Daillere et al. 2016 [67]
human/mouse intratumoral samples	gemcitabine	colon cancer PDAC	The presence of *Mycoplasma hyorhinis* contributed to gemcitabine resistance in the colorectal cancer murine model. Microbiome analysis of tumor samples from PDAC patients revealed that the abundance of *Gammaproteobacteria* was correlating with the resistance to therapy.	Geller et al. 2017 [71]
human/mouse colorectal tissue samples	oxaliplatin 5-FU	colorectal carcinoma	Patient samples showed an association between a higher amount of *Fusobacterium nucleatum* and the promotion of chemoresistance and reduced survival without recurrence. Similarly, the presence of *Fusobacterium nucleatum* eliminated the effect of oxaliplatin in a murine model treated with different doses of oxaliplatin.	Yu et al. 2017 [64]
human feces	chemotherapeutic cocktail containing 5-FU and oxaliplatin	colorectal cancer	A comprehensive analysis of microbial composition in colorectal carcinoma patients treated with chemotherapy revealed the abundance of *Firmicutes* and *Bacteroidetes* phyla. In particular, *Fusobacterium*, *Oscillospira*, and *Prevotella* were presented. Bacterial species *Bacteroides plebeius*, *Veillonella* dispar, and *Prevotella copri* were observed only in fecal samples from patients treated with a conventional chemotherapeutic cocktail.	Deng et al. 2018 [89]
mouse feces	gemcitabine	pancreatic cancer	Decreased levels of *Firmicutes* and *Bacteroidetes* and a higher abundance of *Proteobacteria* and *Verrucomicrobia* were observed in fecal samples from gemcitabine-receiving mice. At the species level, the amounts of *Akkermansia muciniphila* and *Escherichia coli* were significantly increased while the presence of *Bacteroides acidifaciens* was decreased compared to control samples.	Panebianco et al. 2018 [77]
human/mouse feces	variety of cytotoxic targeted chemotherapy immunotherapy	different types of solid tumors and hematological malignancies	An abundance of *Bacteroides ovatus*, *Bacteroides xylanisolvens*, *Prevotella copri*, and *Alistipes* spp. in responder samples correlated with an enhanced response to the therapy. On the contrary, *Clostridium symbiosum * and *Ruminococcus gnavus* were enriched in feces from non-responders. Oral administration of *Bacteroides ovatus/xylanisolvens* into antibiotic pre-treated mice showed a positive impact on reduced tumor growth.	Heshiki et al. 2020 [61]
human feces	neoadjuvant chemotherapy	rectal cancer	Differences in microbiota composition have revealed that non-responder samples were enriched in bacteria belonging to the *Clostridiales* order while patients grouped into responders were characterized by a higher abundance of *Shuttleworthia.*	Shi et al. 2020 [60]

Abbreviations: 5-FU, fluorouracil; PDAC, pancreatic ductal adenocarcinoma; ROS, reactive oxygen species.

**Table 2:** The emerging role of the gut microbiome in efficacy of immunotherapy. The table summarizes the major findings from preclinical and clinical studies.

Types of Samples	Type of Immunotherapy	Malignancy	Major Findings	Study [Ref.]
mouse feces	anti- IL-10R CpG oligonucleotide	colon carcinoma lymphoma melanoma	GF and antibiotic-treated mice reported worse response to therapy. Antibiotics caused decreased production of TNF, reduced survival, and an impaired possibility to retard tumor size. Transfer of bacterial lipopolysacharides into antibiotic-treated animals returned TNF production. Fecal samples revealed that *Alistipes shaii* positively correlated with TNF production.	Iida et al. 2013 [63]
mouse feces	PD-L1 blockade	melanoma	Oral supplementation of *Bifidobacterium* alone helped to eliminate tumor growth after immunotherapy in non-responder tumorigenic mice.	Sivan et al. 2015 [96]
human/mouse feces	ipilimumab	melanoma	The anticancer effect of CTLA-4 in animal models and humans was associated with intestinal composition. It was possible to reconstruct the response to therapy in GF mice via modulation of gut composition by fecal transfer enriched in *Bacteroides* spp. from responder patients.	Vetizou et al. 2015 [117]
human feces	ipilimumab	metastatic melanoma	The results showed the absence of ipilimumab-induced colitis in patients with an abundance of *Bacteroidetes* phylum in analyzed samples.	Dubin et al. 2016 [124]
human feces	ipilimumab nivolumab pembrolizumab a combination of ipilimumab/nivolumab	metastatic melanoma	Feces from pembrolizumab responders were enriched in *Dorea formicigenerans.* On the other hand, *Bacteroides thetaiotamicron*, *Faecalibacterium prausnitzii*, and *Holdemania filiformi* were presented in responders to ipilimumab plus nivolumab therapy.	Frankel et al. 2017 [134]
human feces	ipilimumab	metastatic melanoma	The presence of a higher proportion of *Firmicutes* was associated with benefit from ipilimumab therapy; however, a higher possibility of ipilimumab-induced colitis was observed. On the other hand, the abundance of *Bacteroidetes* correlated with the absence of treatment-related colitis.	Chaput et al. 2017 [118]
human feces	nivolumab	NCSLC	The composition of gut microbiota influenced the efficacy of therapy, showing non-responder samples were enriched in *Dialister*, *Ruminococcus bromii*, *Sutterella*. The abundance of *Akkermansia muciniphila*, *Bifidobacterium longum*, *Faecalibacterium prausnitzii*, *Peptostreptococcus*, *Propionibacterium acnes*, *Staphylococcus aureus*, *Veillonella parvula* was found in responder feces.	Botticelli et al. 2018 [126]
human feces	PD-L1 a combination of PD-L1/CTLA-4	RCC NSCLC	The results confirmed that antibiotic usage decreased the efficacy of immunotherapy. In addition, overall survival and progression-free survival were significantly shortened in antibiotic-treated patients.	Derosa et al. 2018 [132]
human feces	PD-1 blockade	NSCLC gastric cancer	Differences in gut microbiota diversity were documented in patients responding to immunotherapy compared to non-responders. Microbiome analysis of fecal samples from responders revealed the relative abundance of *Ruminococcaceae* family.	Fukuoka et al., 2018 [125]
human/mouse feces	PD-1 blockade	melanoma	The high levels of *Faecalibacterium* species were found in responders. Non-responders were characterized by the presence of *Anaerotruncus colihominis*, *Bacteroides thetaiotaomicron*, and *Escherichia coli.* FMT from responders into recipient GF animals led to hindered tumor growth and mouse recipients reported a higher abundance of *Faecalibacterium* in their gut microbiome.	Gopalakrishnan et al. 2018 [122]
human feces	nivolumab	RCC	The stool samples from responders to immune checkpoint blockade were relatively abundant in *Roseburia* and *Faecalibacterium spp*.	Maia et al. 2018 [127]
human feces	PD-1/PD-L1 blockade	metastatic melanoma	Responders were enriched in *Bifidobacterium adolescentis*, *Bifidobacterium longu*, *Collinsella aerofaciens*, *Enterococcus faecium*, *Klebsiella pneumoniae*, *Parabacteroides merdae*, *Veillonella parvula.* Importantly, the transfer of fecal samples led to the anticancer response in GF mice.	Matson et al. 2018 [123]
human/mouse feces	PD-1/PD-L1 blockade	NSCLC urothelial carcinoma RCC	Responder fecal samples were enriched in *Akkermansia muciniphila*, According to the findings from animal models, GF recipients of FMT from non-responders showed higher efficacy of immunotherapy after supplementation with *Akkermansia muciniphila*.	Routy et al. 2018 [119]
human feces	PD-1/CTLA-4	metastatic melanoma	A higher intestinal richness was connected with longer progression-free survival and a low risk of progression was associated with the presence of *Coprococcus eutactus*, *Faecalibacterium prausnitzii*, *Lachnospiraceae bacterium 3 1 46FAA*, *Prevotella stercorea*, *Streptococcus anginosus*, and *Streptococcus sanguinis.*	Peters et al. 2019 [130]
human feces	PD-1 blockade	hepatocellular carcinoma	Responder samples showed a higher taxa diversity, enriched in *Akkermansia muciniphila* and *Ruminococcaceae* spp. while non-responder samples were abundant mainly in *Escherichia coli* belonging to *Proteobacteria* phylum.	Zheng et al. 2019 [128]
human/mouse feces	nivolumab	advanced RCC	*Akermansia muciniphila* and *Bacteroides salyersiae* were presented in non-primary resistant patient samples. FMT from non-resistant patients into resistant mice restored the response to nivolumab therapy.	Derosa et al., 2020 [133]
mouse feces	CTLA-4 blockade	colorectal carcinoma	Specific bacterial species including *Bifidobacterium pseudolongum*, *Lactobacillus johnsonii*, and *Olsenella spp*., presented in monocolonized mouse model, increased the efficacy of immunotherapy compared to monocolonization with *Colidextribacter species* or *Prevotella species*.	Mager et al. 2020 [121]
human feces	nivolumab a combination of ipilimumab/nivolumab	metastatic RCC	A higher gut diversity, with the prevalence of *Akkermansia muciniphila*, enhanced the benefit from immune checkpoint blockade in patients.	Salgia et al. 2020 [129]
mouse feces	PD-1 blockade	colorectal carcinoma	Altered gut microbiota led to metabolic changes. In a mouse model, the presence of *Akkermansia muciniphila* and *Prevotella* spp. improved the efficacy of immunotherapy.	Xu et al. 2020 [120]
human feces	nivolumab	metastatic melanoma	Donor stool samples enriched in *Lachnospiraceae*, *Ruminococcaceae*, and *Veillonellaceae* were used for FMT in refractory patients. Subsequently, a response to antitumor therapy was detected in some of the patients.	Baruch et al. 2021 [135]
human feces	pembrolizumab	metastatic melanoma	Donor fecal samples were transferred into refractory metastatic melanoma patients. After FMT, responder recipient samples shared the composition of microbial community with donor samples. Available data showed that gut microbiota was enriched in *Actinobacteria* and *Firmicutes* while *Bacteroidetes* were found to be reduced.	Davar et al. 2021 [136]

Abbreviations: GF, germ-free; FMT, fecal microbiota transplantation; NSCLC, non-small cell lung cancer; RCC, renal cell carcinoma; TNF, tumor necrosis factor.
